# Calcium Imaging Analysis of Cellular Responses to Hypercapnia and Hypoxia in the NTS of Newborn Rat Brainstem Preparation

**DOI:** 10.3389/fphys.2021.645904

**Published:** 2021-03-25

**Authors:** Hiroshi Onimaru, Itaru Yazawa, Kotaro Takeda, Isato Fukushi, Yasumasa Okada

**Affiliations:** ^1^Department of Physiology, Showa University School of Medicine, Tokyo, Japan; ^2^Global Research Center for Innovative Life Science, Hoshi University School of Pharmacy and Pharmaceutical Sciences, Tokyo, Japan; ^3^Faculty of Rehabilitation, School of Healthcare, Fujita Health University, Toyoake, Japan; ^4^Faculty of Health Sciences, Uekusa Gakuen University, Chiba, Japan; ^5^Clinical Research Center, Murayama Medical Center, Musashimurayama, Japan

**Keywords:** NTS, astrocyte, hypoxia, hypercapnia, calcium imaging, rat, isolated brainstem-spinal cord preparation

## Abstract

It is supposed that the nucleus of the solitary tract (NTS) in the dorsal medulla includes gas sensor cells responsive to hypercapnia or hypoxia in the central nervous system. In the present study, we analyzed cellular responses to hypercapnia and hypoxia in the NTS region of newborn rat *in vitro* preparation. The brainstem and spinal cord were isolated from newborn rat (P0-P4) and were transversely cut at the level of the rostral area postrema. To detect cellular responses, calcium indicator Oregon Green was pressure-injected into the NTS just beneath the cut surface of either the caudal or rostral block of the medulla, and the preparation was superfused with artificial cerebrospinal fluid (25–26°C). We examined cellular responses initially to hypercapnic stimulation (to 8% CO_2_ from 2% CO_2_) and then to hypoxic stimulation (to 0% O_2_ from 95% O_2_ at 5% CO_2_). We tested these responses in standard solution and in two different synapse blockade solutions: (1) cocktail blockers solution including bicuculline, strychnine, NBQX and MK-801 or (2) TTX solution. At the end of the experiments, the superfusate potassium concentration was lowered to 0.2 from 3 mM to classify recorded cells into neurons and astrocytes. Excitation of cells was detected as changes of fluorescence intensity with a confocal calcium imaging system. In the synaptic blockade solutions (cocktail or TTX solution), 7.6 and 8% of the NTS cells responded to hypercapnic and hypoxic stimulation, respectively, and approximately 2% of them responded to both stimulations. Some of these cells responded to low K^+^, and they were classified into astrocytes comprising 43% hypercapnia-sensitive cells, 56% hypoxia-sensitive cells and 54% of both stimulation-sensitive cells. Of note, 49% of the putative astrocytes identified by low K^+^ stimulation were sensitive to hypercapnia, hypoxia or both. In the presence of a glia preferential blocker, 5 mM fluoroacetate (plus 0.5 μM TTX), the percentage of hypoxia-sensitive cells was significantly reduced compared to those of all other conditions. This is the first study to reveal that the NTS includes hypercapnia and hypoxia dual-sensitive cells. These results suggest that astrocytes in the NTS region could act as a central gas sensor.

## Introduction

The nucleus of the solitary tract (NTS) located in the dorsal medulla is the first relay station of sensory inputs involving autonomic functions and works as an integrative system that processes these inputs. Indeed, there are many reports that the NTS plays a role in controlling sympathetic activity and eupneic breathing (Andresen and Kunze, 1994; Braga et al., 2007; Subramanian et al., 2007; Braccialli et al., 2008; Abdala et al., 2009; Accorsi-Mendonca et al., 2011; Costa et al., 2013). Moreover, it is supposed that the NTS includes gas sensor cells responsive to hypercapnia and/or hypoxia in the central nervous system. It is known that the NTS region contributes to the regulation of the hypercapnic ventilatory response (Nattie and Li, 2002, 2008, 2009). Indeed, CO_2_/H^+^ chemosensitive neurons were found in various subnuclei of the NTS (Dean et al., 2001; Nichols et al., 2008; Dean and Putnam, 2010; Huda et al., 2012). Recent studies showed that a subgroup of Phox2b-expressing neurons in the NTS exhibited intrinsic chemosensitivity to hypercapnic stimulation (Fu et al., 2017) and was presumed to participate in the hypercapnic ventilatory response (Fu et al., 2019).

There is significant evidence that astrocytes in the ventral medulla are involved in central chemosensory mechanisms that maintain cardiorespiratory homeostasis (Gourine et al., 2010; Marina et al., 2016; Turovsky et al., 2016). Systemic hypercapnia, which leads to decreases in blood and brain pH, is associated with a rapid release of ATP within the ventral chemosensory areas of the brainstem (Gourine et al., 2005a). It was also suggested that astrocytes in the NTS contribute to the CO_2_/H^+^ response by affecting synaptic transmission (Huda et al., 2013). In the ventral medulla, ATP-dependent mechanism to regulate respiratory activity is also involved in hypoxic response (Gourine et al., 2005b; Rajani et al., 2017). In the dorsal medulla, Tadmouri et al. (2014) suggested that astrocytes in the NTS contributed to the neuronal response during the first hour of hypoxia. Enhanced firing in the NTS neurons induced by short-term sustained hypoxia was modulated by glia-neuron interaction (Accorsi-Mendonca et al., 2015). Astrocytic modulation of glutamatergic synaptic transmission was reduced in the NTS of rats submitted to short-term sustained hypoxia (Accorsi-Mendonca et al., 2019). In contrast, acute inhibition of glial cells by bilateral microinjections of fluorocitrate in the NTS did not affect respiratory or sympathetic activities in rats exposed to chronic intermittent hypoxia (Costa et al., 2013).

Thus, cells in the NTS are presumed to be involved directly or indirectly in responses to hypercapnia or hypoxia. However, no study has investigated detailed structures of cell components in the responses, i.e., hypercapnia sensitive, hypoxia sensitive or both types. To answer this question, we analyzed cellular responses to hypercapnia and hypoxia in the NTS region of newborn rat *in vitro* preparation by multi-cell recordings using calcium imaging.

## Materials and Methods

**FIGURE 1:**
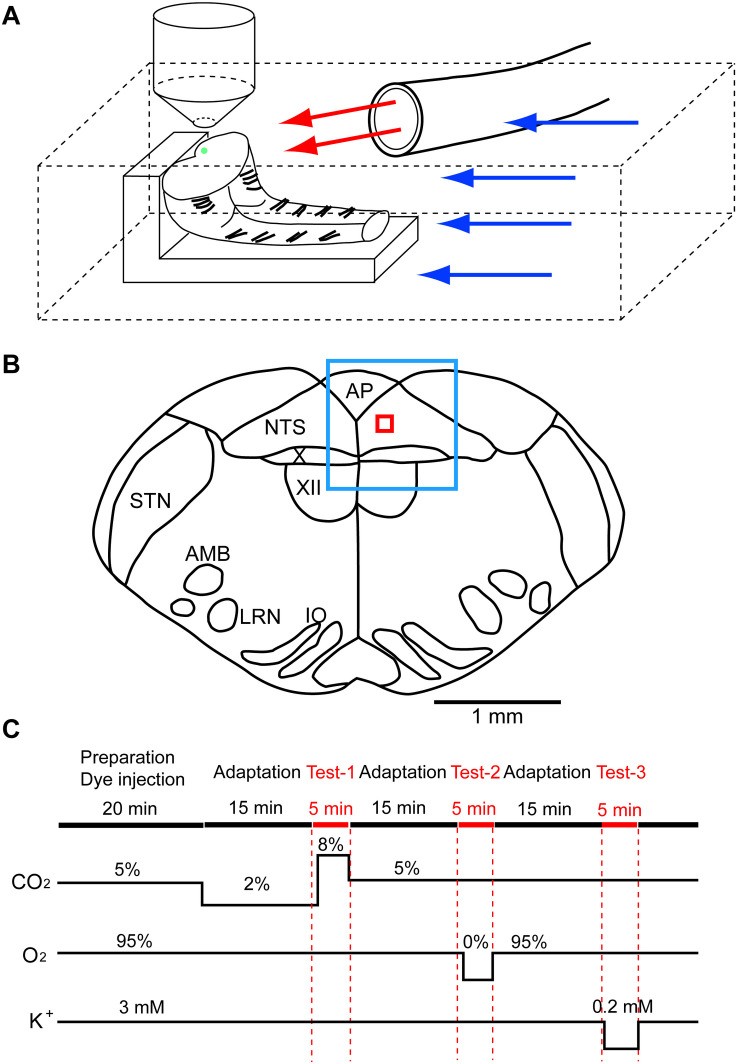
Setup of preparation and rapid exchange system of superfusate and an approximate region of fluorescence recording. **(A)** The preparation (in this case, caudal block) was set on silicon rubber with the cut surface facing up. Artificial cerebrospinal fluid was superfused through two lines, (1) a main route perfusing the experimental chamber (blue arrows) and (2) a sub-route (red arrows) through a glass tube (inside diameter, 2 mm) that was placed close to the preparation. Under the control condition, the control solution was perfused through both routes. The test solution was applied to the preparation via the sub-route by replacing the control solution with the test solution. This system allowed rapid exchange (within 30 s) of the test solution. The green dot on the cut surface denotes an approximate injection area of the calcium indicator Oregon Green. **(B)** A typical cut surface of the medulla and an approximate region of the recording. The blue square denotes the focused area at low magnification (4×), and the red square denotes the recording area of fluorescence intensity at high magnification (40×). AMB, nucleus ambiguus; AP, area postrema; IO, inferior olivary nucleus; LRN, lateral reticular nucleus; NTS, nucleus of the tractus solitarius; STN, spinal trigeminal nucleus; X, dorsal motor nucleus of the vagus; XII, hypoglossal nucleus. **(C)** Protocol of experiments. Red marks denote periods of calcium imagings: Test-1, hypercapnic stimulation; Test-2, hypoxic stimulation; Test-3, low K^+^ stimulation.

The experimental protocols were approved by the Ethics Committee for Animal Experiments of Murayama Medical Center. The brainstem and spinal cord were isolated together from newborn rat (Wistar, P0–P4) under deep isoflurane anesthesia and were transversely cut at the level of the rostral area postrema with a custom-made vibratome. From a single brainstem-spinal cord, two preparations were obtained, a rostral block of the medulla and a caudal block of the medulla-spinal cord, and used for recordings. The preparation was set on silicon rubber with the cut surface facing up (Figure 1A). To record cellular activities, a calcium indicator, Oregon Green 488 BAPTA-1 AM (200 μM; Invitrogen, Carlsbad, CA), was pressure-injected into the NTS just beneath the cut surface of either the rostral or caudal block preparation (Figure 1B), and the preparation was superfused with artificial cerebrospinal fluid (ACSF) composed of the following (in mM): 118 NaCl, 3 KCl, 1 CaCl_2_, 1 MgCl_2_, 26 NaHCO_3_, 1.2 NaH_2_PO_4_ and 30 glucose equilibrated with 95% O_2_ and 5% CO_2_, pH 7.4, at 25–26°C (Okada et al., 2012). We examined cellular responses initially to hypercapnic stimulation (to 8% CO_2_ from 2% CO_2_) and then to hypoxic stimulation (to 0% O_2_ from 95% O_2_ at 5% CO_2_) (Figure 1C). We then tested these responses in standard ACSF and two different synapse blockade solutions including (1) 5 μM (-)-bicuculline methiodide (Sigma-Aldrich, Tokyo, Japan), 5 μM strychnine sulfate (FUJIFILM Wako Pure Chemical, Osaka, Japan), 2 μM NBQX (Sigma-Aldrich) and 10 μM (+)-MK-801 maleate (Tocris, Funakoshi Co., Tokyo, Japan) and (2) 0.5 μM TTX (Latoxan Laboratory, Portes-lès-Valence, France). In some experiments, responses to hypercapnia and hypoxia were examined in the presence of glia preferential blocker fluoroacetate (FA; Hülsmann et al., 2000) (FUJIFILM Wako Pure Chemical). At the end of the experiments, the superfusate potassium concentration was lowered from 3 to 0.2 mM to classify recorded cells into neurons and astrocytes because lowered potassium induces vigorous rises in intracellular calcium in astrocytes but not in neurons (Dallwig et al., 2000; Dallwig and Deitmer, 2002; Hartel et al., 2007). Preparations were pre-incubated for 15 min in the pre-stimulus solution (2% CO_2_ or 2% CO_2_ + blockers) before application of the first test solution (Figure 1C). Each measurement was separated by an interval of least 10–15 min to allow wash-out. The Oregon Green dye is pH insensitive in the physiological range (Invitrogen Technical Information Sheet). Therefore, changes in the fluorescence intensity during hypercapnic stimulation (2–8% CO_2_ corresponding to pH 7.8–7.2) would not be disturbed by the pH-dependent property of the dye itself.

**FIGURE 2:**
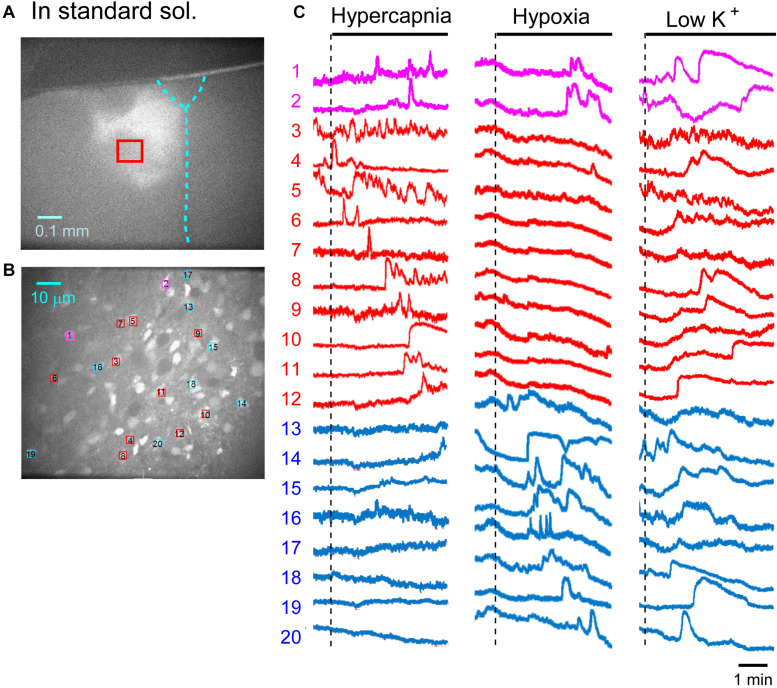
Calcium imaging in the NTS in standard solution (sol.). **(A)** Low-magnification image of the cut surface where Oregon Green was injected and calcium imaging was performed. **(B)** Optical image of the cut surface stained with Oregon Green. The red square in **(A)** corresponds to the measured area shown in **(B)**. **(C)** Calcium signals plotted as fluorescence intensity in identified NTS cells numbered in the image in **(B)** in response to hypercapnia (left), hypoxia (middle) and low K^+^ (right) stimulation. Magenta traces denote calcium signal intensity of cells (Nos. 1 and 2) responding to stimulation of both hypercapnia and hypoxia. Red traces denote calcium signal intensity of cells (Nos. 3–12) responding only to hypercapnia. Blue traces denote calcium signal intensity of cells (Nos. 13–20) responding only to hypoxia.

Superfusing ACSF was supplied via two routes: (1) a main route perfusing the entire recording chamber (blue arrows in Figure 1A) and (2) a sub-route (red arrows) through a glass tube (inside diameter, 2 mm) that was placed close to the preparation. Under the control condition, the control solution was perfused through both routes. The test solution was applied to the preparation via the sub-route by replacing the control solution with the test solution. The rate of superfusion was set at 2–3 mL/min in each route. This system allowed rapid application (within 30 s) of the test solution to the preparation.

The cell-bound calcium indicator dye in the NTS was excited by a 488-nm laser beam using a laser diode (Cobolt 06-MLD, HÜBNER Photonics, Kassel, Germany), and cellular activities were visualized through a 520-nm long-pass emission filter. Images were captured every 0.3 s for 5 min in each measurement using a Nipkow-disk confocal scanner unit (CSU21; Yokogawa Electric, Tokyo, Japan), an electron-multiplying CCD camera (Luca S 658M; Andor Technology, Belfast, United Kingdom), an upright fluorescent microscope (Eclipse E600FN; Nikon, Tokyo, Japan) and a water-immersion objective lens (40×, 0.8 NA, Fluor, Nikon). Figure 1B shows an approximate region of the recording: the blue square denotes a focused area at low magnification (4×), and the red square denotes a recording area of fluorescence intensity at high magnification (40×). Test solutions (hypercapnia or hypoxia) were applied at 1 min after the start of data acquisition in each measurement. Low K^+^ solution was applied at 30 s after the start of data acquisition. Mean fluorescence intensity at 3.5 × 3.5 μm^2^ of the region of interest (ROI) was calculated by the software Andor SOLIS for Imaging (Oxford Instruments plc, Abingdon, United Kingdom). Cells responding to stimulation were detected by rapid change (typically more than 5% within less than 5 s) of the brightness in a cell within the ROI. We calculated ΔF/F of the peak value, which is the ratio of difference of the fluorescence intensity against that of the baseline (average of 10 frames) immediately before the peak. The time taken for fluorescence intensity to increase compared with the baseline (duration of excitation) was also calculated. The first 50 frames (15 s) were removed because of a light-intensity settling artifact. We made an effort to minimize laser illumination time throughout the experiments to avoid a photobleaching effect.

We counted the number of cells that responded to stimulation. Statistical examination of the percentage of responding cells was evaluated by chi-square test (Microsoft Excel) at a confidence level of *P* < 0.05. Data such as peak values are presented as means ± SD, and the significance of the values was analyzed by one-way ANOVA, followed by a Tukey–Kramer multiple comparisons test at a confidence level of *P* < 0.05 using the GraphPad InStat software program (GraphPad Software, La Jolla, CA, United States).

## Results

### Responses in the Standard Solution or Synapse Blockers

**FIGURE 3:**
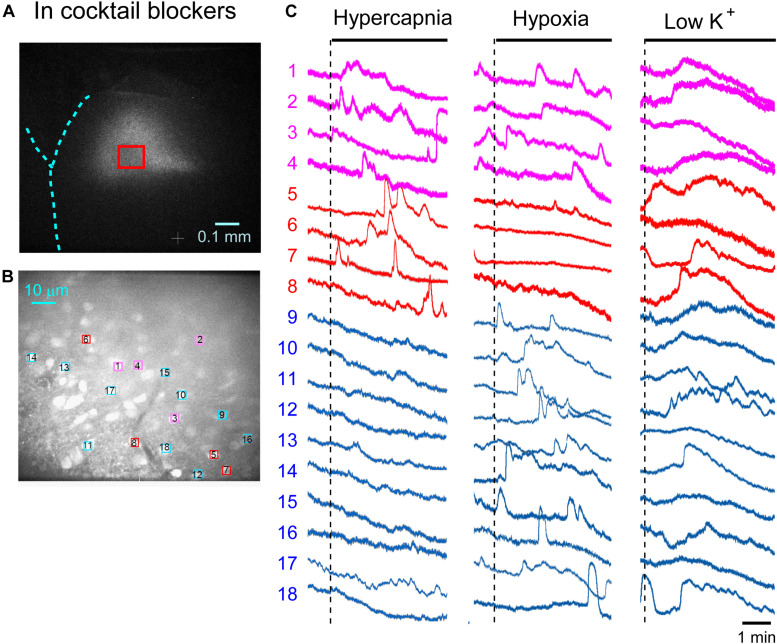
Calcium imaging in the NTS in cocktail blockers solution. **(A)** Low-magnification image of the cut surface where Oregon Green was injected and calcium imaging was performed. **(B)** Optical image of the cut surface stained with Oregon Green. The red square in **(A)** corresponds to the measured area shown in **(B)**. **(C)** Calcium signals plotted as fluorescence intensity in identified NTS cells numbered in the image in **(B)** in response to hypercapnia (left), hypoxia (middle) and low K^+^ (right) stimulation. Magenta traces denote calcium signal intensity of cells (Nos. 1–4) responding to stimulation of both hypercapnia and hypoxia. Red traces denote calcium signal intensity of cells (Nos. 5–8) responding only to hypercapnia. Blue traces denote calcium signal intensity of cells (Nos. 9–18) responding only to hypoxia.

**FIGURE 4:**
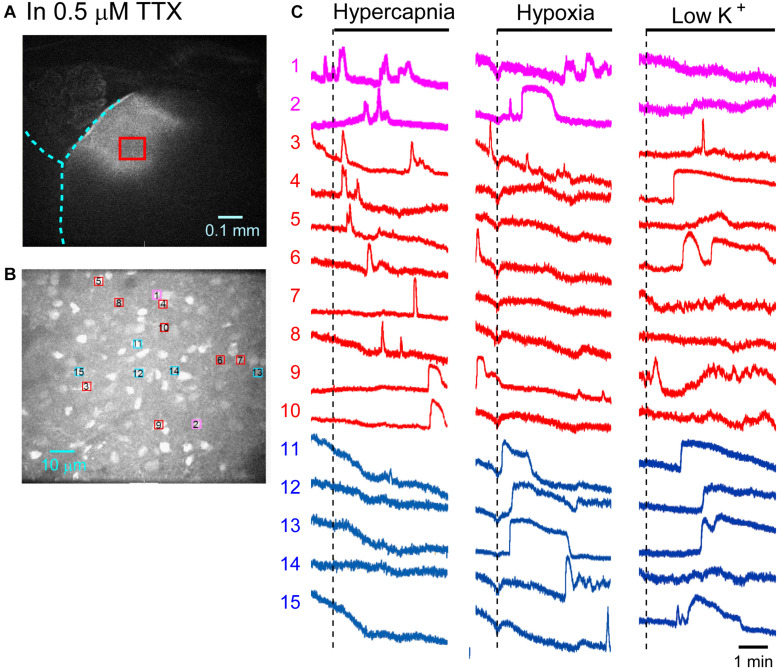
Calcium imaging in the NTS in TTX solution. **(A)** Low-magnification image of the cut surface where Oregon Green was injected and calcium imaging was performed. **(B)** Optical image of the cut surface stained with Oregon Green. The red square in **(A)** corresponds to the measured area in **(B)**. **(C)** Calcium signals plotted as fluorescence intensity in identified NTS cells numbered in the image in **(B)** in response to hypercapnia (left), hypoxia (middle) and low K + (right) stimulation. Magenta traces denote calcium signal intensity of cells (Nos. 1 and 2) responding to stimulation of both hypercapnia and hypoxia. Red traces denote calcium signal intensity of cells (Nos. 3–10) responding only to hypercapnia. Blue traces denote calcium signal intensity of cells (Nos. 11–15) responding only to hypoxia.

First, we examined cellular responses in the standard ACSF (Figure 2). In this example, 12 cells (Nos. 1–12) responded to hypercapnic stimulation and 10 cells (Nos. 1, 2, 13–20) responded to hypoxic stimulation. Two of these cells (Nos. 1 and 2) responded to both stimulations, and 14 of these cells responded to low K^+^ stimulation (Supplementary Video 1). The activity pattern was rather complex, and the duration of excitation was variable (4–160 s); some cells showed a relatively short transient increase with one or several peaks (e.g., trace 1 or 2 in Figure 2C Hypercapnia) and others showed a long-lasting plateau-like increase (e.g., trace 8 or 10 in Figure 2C Hypercapnia). We counted the number of cells that responded to stimulation regardless of the activity pattern. Results from 5 preparations are shown in Table 1A. Next, we examined cellular responses in the synaptic blockade ACSF (cocktail blockers solution, Figure 3). In this example, 8 cells (Nos. 1–8) responded to hypercapnic stimulation, and 14 cells (Nos. 1–4, 9–18) responded to hypoxic stimulation. Four of these cells (Nos. 1–4) responded to both stimulations, and 8 cells responded to low K^+^ stimulation. Results from 5 preparations are shown in Table 1B. Then, we examined cellular responses in the presence of TTX (0.5 μM TTX solution, Figure 4). In this example, 10 cells (Nos. 1–10) responded to hypercapnic stimulation, and 7 cells (Nos. 1, 2, 11–15) responded to hypoxic stimulation. Two of these cells (Nos. 1 and 2) responded to both stimulations, and 8 cells responded to low K^+^ stimulation. Results from 5 preparations are shown in Table 1C.

**TABLE 1:** Summary of number of responding cells and total cell number.

	N	% (1)	Low K^+^	% (2)
**A. In Standard sol (5 preparations)**				
Hypercapnia	49	10.5	27	55.1
Hypoxia	39	8.4	25	64.1
Both	11	2.4	9	81.8
Sum	77	16.5	42	54.5
Total cell number	467		89	19.1
**B. In cocktail (5 preparations)**				
Hypercapnia	38	7.9	17	44.7
Hypoxia	40	8.4	22	55.0
Both	12	2.7	6	50.0
Sum	66	13.8	33	50.0
Total cell number	479		67	14.0
**C. In TTX (5 preparations)**				
Hypercapnia	34	7.3	14	41.2
Hypoxia	35	7.5	20	57.1
Both	7	1.5	4	57.1
Sum	62	13.3	30	48.4
Total cell number	466		95	20.4
**D. In fluoroacetate (5 preparations)**				
Hypercapnia	24	5.2	6	25.0
Hypoxia	18	3.9	7	38.9
Both	6	1.3	2	33.3
Sum	36	7.9	11	30.6
Total cell number	458		57	12.4

*Hypercapnia, cells that responded to hypercapnic stimulation (2% CO_2_→8% CO_2_); Hypoxia, cells that responded to hypoxic stimulation (95% O_2_→0% O_2_); Both, cells that responded to both hypercapnic and hypoxic stimulations; Total cell number, sum of cell number from 5 preparations in the visual field of fluorescence measurement; N, number of cells;% (1), percentage of responding cells to total cell number; Low K^+^, number of cells responding to low K^+^ stimulation of the cells that responded to each stimulation; Total cell number of Low K^+^, total cell number of low K^+^-responding cells that includes cells that did not respond to the gas stimulation;% (2), percentage of low K^+^-responding cells of the cells that responded to gas stimulation;% (2) of Total cell number, percentage of low K^+^-responding cells to total cell number; A, in standard solution; B, in cocktail blockers solution; C, in 0.5 μM TTX solution and D, in 5 mM fluoroacetate + 0.5 μM TTX solution. The red color indicates values whose statistical significance was evaluated (see text).*

Based on the total cell number from 5 preparations of each condition (Table 1), the percentages of responding cells were calculated. In the standard ACSF (control solution), 10.5% of the NTS cells responded to hypercapnic stimulation, 8.4% responded to hypoxic stimulation, and 2.4% responded to both stimulations. In the synaptic blockade solutions (cocktail or 0.5 μM TTX solutions), the number of NTS cells responding to hypercapnic stimulation tended to decrease, but not significantly so. Thus, similar percentages of hypercapnia- and/or hypoxia-responding cells were observed regardless of whether the synaptic transmission was intact or blocked. Some of these cells responded to low K^+^, and thus they were classified into astrocytes: 43% of the hypercapnia-sensitive cells, 56% of the hypoxia-sensitive cells and 54% of both stimulation-sensitive cells under a synaptic blockade condition (i.e., cocktail and TTX solution). Of note, 49% of the putative astrocytes identified by low K^+^ stimulation were sensitive to hypercapnia, hypoxia or both.

### Responses in the Presence of a Glia Preferential Blocker

**FIGURE 5:**
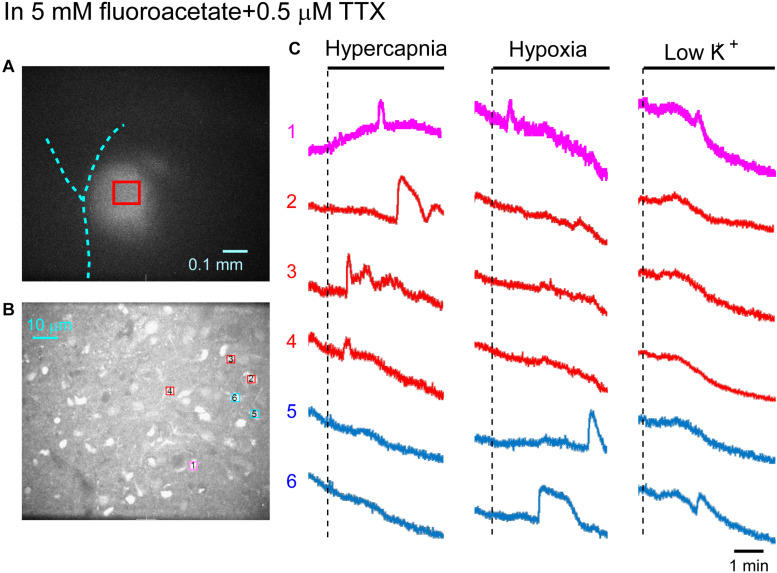
Calcium imaging in the NTS in fluoroacetate plus TTX solution. **(A)** Low-magnification image of the cut surface where Oregon Green was injected and calcium imaging was performed. **(B)** Optical image of the cut surface stained with Oregon Green. The red square in **(A)** corresponds to measured area in **(B)**. **(C)** Calcium signals plotted as fluorescence intensity in identified NTS cells numbered in the image in **(B)** in response to hypercapnia (left), hypoxia (middle) and low K^+^ (right) stimulation. Magenta trace denotes calcium signal intensity of cells (No. 1) responding to stimulation of both hypercapnia and hypoxia. Red traces denote calcium signal intensity of cells (Nos. 2–4) responding only to hypercapnia. Blue traces denote calcium signal intensity of cells (Nos. 5 and 6) responding only to hypoxia.

**FIGURE 6:**
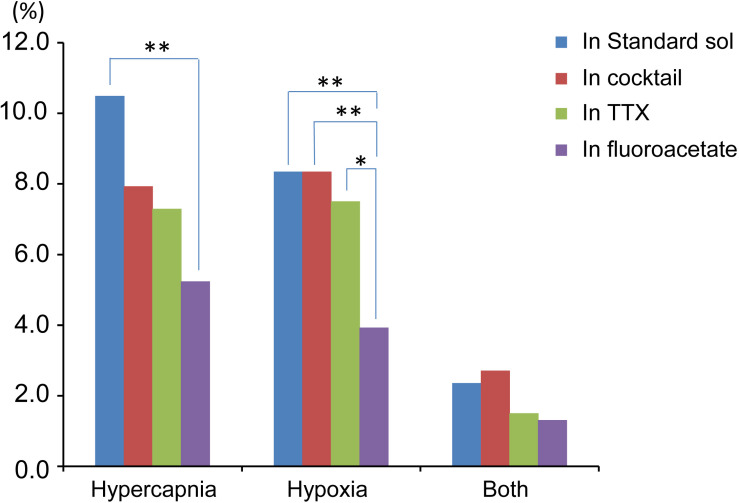
Summary of percentage of responding cells to total cell number based on Table 1. Hypercapnia, cells that responded to hypercapnic stimulation (2% CO_2_→8% CO_2_). Hypoxia, cells that responded to hypoxic stimulation (95% O_2_→0% O_2_). Both, cells that responded to both hypercapnic and hypoxic stimulations. Each bar indicates different extracellular conditions: standard solution (sol), cocktail blockers solution, 0.5 μM TTX solution and 5 mM fluoroacetate + 0.5 μM TTX solution. ^∗^*P* < 0.05, ^∗∗^*P* < 0.01.

In the next step, we examined cellular responses in the presence of a glia preferential blocker, FA. A typical example is shown in Figure 5. In the presence of 5 mM FA (plus 0.5 μM TTX), the percentage of hypercapnia-sensitive cells was significantly lower compared to that in the standard solution (*P* < 0.01). The percentage of hypoxia-sensitive cells was significantly reduced compared to those of all other conditions (Figure 6 and Table 1), and that of low K^+^-responding cells in FA (+ TTX) solution was also significantly lower than that in TTX solution without FA (*P* < 0.01).

When we analyzed the duration of excitation (Supplementary Table 1), it tended to be longest during low K^+^ stimulation and shortest during hypercapnic stimulation. There was no significant difference in the averaged peak amplitude (ΔF/F) within each experimental condition (standard, cocktail, TTX and FA in Supplementary Table 1). However, ΔF/F tended to be lower in the TTX and FA solutions when values in the same stimulus condition (i.e., hypercapnia, hypoxia and low K^+^) were compared.

Our experimental protocol was performed sequentially: first hypercapnia, next hypoxia and finally low K^+^ test. Therefore, responses to hypoxia and low K^+^ might be affected by the preceding test and elapsed time. To evaluate these effects, we conducted subsequent additional tests: initially hypoxia and then the low K^+^ test in the absence (*n* = 7) and presence (*n* = 5) of TTX. The percentage of cells responding to hypoxia was comparable to the case in which hypercapnia tests were performed first. However, the percentage of low K^+^-responding cells tended to decrease in the case in which hypercapnia was followed by hypoxia prior to low K^+^ testing (Table 2), suggesting that some astrocytes might not be responding to low K^+^.

**TABLE 2:** Effects of preceding treatments on responses to hypoxic stimulation and low K^+^ stimulation.

	Hypoxia → Low K^+^		Hypercapnia →		
			Hypoxia → Low K^+^		
		
	Standard	TTX	Standard	Cocktail	TTX
Hypoxia (%)	9.9	9.5	8.4	9.8	7.9
Low K^+^ (%) to responding cells	66.2	56.8	54.5	38.3	54.1
Low K^+^ (%) to total	23.7	23.8	19.1	13.6	20.4

*Hypoxia→Low K^+^, initially hypoxic stimulation (95% O_2_→0% O_2_) was tested and then low K^+^ was tested; Hypercapnia→Hypoxia→Low K^+^, initially hypercapnic stimulation (2% CO_2_→8% CO_2_) was tested and then hypoxia and low K^+^ were tested; Standard, in standard solution; TTX, in 0.5 μM TTX solution; Cocktail, in cocktail blockers solution; Hypoxia (%), percentage of cells that responded to hypoxic stimulation; Low K^+^ (%) to responding cells, percentage of low K^+^-responding cells of the cells that responded to hypoxic stimulation; Low K^+^ (%) to total, percentage of low K^+^-responding cells to total cell number.*

## Discussion

We investigated cellular responses in the NTS during the first 4 min after hypercapnic or hypoxic stimulation by calcium imaging. We found that the NTS included cells that were sensitive to hypercapnia or hypoxia and some that were sensitive to both stimulations. Furthermore, some of the cells were identified as astrocytes by low K^+^ stimulation (Dallwig et al., 2000; Dallwig and Deitmer, 2002; Hartel et al., 2007). The activity pattern of the responding cells varied. The duration of excitation tended to differ between the types of stimulation: hypercapnia < hypoxia < low K^+^. The peak fluorescence intensity (ΔF/F) also tended to differ between the experimental solutions and was lower in the TTX and FA solutions. In the presence of a glia preferential blocker (FA), the percentage of cells that responded to hypoxic stimulation was significantly reduced compared with those in standard solution, cocktail blocker solution and TTX solution. In contrast, the percentage of cells that responded to hypercapnic stimulation was significantly reduced compared with that only in the standard solution. Moreover, FA treatment significantly reduced the percentage of cells that responded to low K^+^ stimulation. Thus, our findings suggest that the contribution of astrocytes as gas sensors may be larger in hypoxia than in hypercapnia, whereas time dependent effects of FA treatment should be considered.

FA and its toxic metabolite fluorocitrate cause inhibition of aconitase. In brain tissue, both substances are preferentially taken up by glial cells and lead to reversible dysfunction of astrocytes with unimpaired neuronal function in the usage of appropriate doses (Clarke, 1991; Swanson and Graham, 1994; Fonnum et al., 1997; Lian and Stringer, 2004; Erlichman and Leiter, 2010). We applied FA totally for 60 min, i.e., for 20 min until the end of hypercapnic stimulation, 40 min until the end of hypoxic stimulation and 60 min until the end of low K^+^ stimulation. Hülsmann et al. (2000) reported that in experiments using the medullary slice preparation, respiratory rhythm could be restored by application of isocitrate or glutamine even after 60 min incubation with 5 mM FA. Therefore, the depression of cell activity induced by FA was probably not due to irreversible cell damage.

Although lowering the extracellular K^+^ concentration hyperpolarizes neurons and astrocytes, it is suggested that Ca^2+^ would enter into astrocytes via Kir4.1 channels during low K^+^ stimulation (Hartel et al., 2007). Therefore, cells that responded to low K^+^ were classified into astrocytes (Dallwig et al., 2000; Dallwig and Deitmer, 2002; Hartel et al., 2007). However, the number of putative astrocytes identified in the present study might have been underestimated because some astrocytes might not have responded to low K^+^ due to the possibly exhausting effects of the preceding tests and elapsed time. Thus, although we could not conclude that cells that did not respond to low K^+^ were neurons, we could conclude that cells that responded to low K^+^ were astrocytes and not neurons because neurons should not be excited by low K^+^ stimulation (Okada et al., 2005).

There are CO_2_/H^+^ chemosensitive neurons in the NTS (Dean et al., 2001; Nichols et al., 2008; Dean and Putnam, 2010; Huda et al., 2012). The transcription factor Phox2b is one of the genetic markers of CO_2_-sensitive neurons in the rostral ventrolateral medulla (Stornetta et al., 2006; Dubreuil et al., 2008; Guyenet et al., 2008; Onimaru et al., 2008). Some chemosensitive neurons in the NTS also express Phox2b (Fu et al., 2017). In the present study, therefore, hypercapnia-sensitive cells in the NTS might include Phox2b-positive neurons. A calcium imaging study of EYFP-expressing Phox2b-positive neurons was performed in the parafacial region (Onimaru et al., 2018), and this method would be applicable to Phox2b-positive cells in the NTS.

We found that some of the cells (including putative astrocytes) responded to stimulation of both hypercapnia and hypoxia, although the intracellular mechanisms were unknown. It is known that hypercapnic stimulation induced the closing of potassium channels and then depolarized neurons in the ventral medulla (Guyenet et al., 2008; Onimaru et al., 2008, 2012; Kumar et al., 2015) and in the NTS (Huda et al., 2012; Fu et al., 2017). It is also reported that in medullary astrocytes, lowering of pH-activated Na^+^/HCO_3_^–^ cotransport raised intracellular Na^+^. Elevation of intracellular Na^+^ activated the Na^+^/Ca^2+^ exchanger, thus inducing Ca^2+^ entry (Turovsky et al., 2016). In contrast, the ionic mechanism in hypoxia response is not well understood. The TRPA1 channel at least partly contributes to cellular responses to moderate hypoxia (Mori et al., 2017; Uchiyama et al., 2020). However, further study is needed to clarify the contribution of the TRPA1 channel mechanism to the hypoxia response of NTS cells.

### Functional Consideration

Several previous studies suggested that astrocytes in the NTS play a role in hypoxic responses (Tadmouri et al., 2014; Accorsi-Mendonca et al., 2015, 2019). In contrast, Costa et al. (2013) showed that acute inhibition of glial cells by bilateral microinjections of fluorocitrate into the NTS did not affect respiratory or sympathetic activities in rats exposed to chronic intermittent hypoxia. However, this finding does not mean that glia cells in the NTS do not contribute to the acute hypoxic response. Huda et al. (2013) reported that acidification-dependent regulation of glial function affects synaptic transmission within the NTS. They suggest that glia play a modulatory role in the NTS by integrating local tissue signals (such as pH) with synaptic inputs from peripheral afferents. The present results suggest that astrocytes in the NTS region could play a role as a central gas sensor, although the physiological function remains to be elucidated.

Previous studies have proposed a presence of central hypoxic sensors that are involved in respiratory response to hypoxia (Gourine and Funk, 2017; Funk and Gourine, 2018). In the ventral medulla, preBötzinger Complex astrocytes contribute to hypoxia sensing and biphasic hypoxic ventilator response, independent of activation of peripheral chemoreceptors (Gourine et al., 2005b; Angelova et al., 2015; Rajani et al., 2017). The physiological relevance of the biphasic hypoxic response in different types of preparations is debatable due to difference of experimental conditions (Funk and Gourine, 2018; Teppema, 2018). In the en bloc preparation, the change of respiratory rhythm during hypoxic stimulation is typically biphasic, with initial augmentation followed by a decline (Okada et al., 1998; see also Supplementary Figure 1). Although the biphasic respiratory response patterns in unanesthetized peripheral-chemodenervated *in vivo* animals and the en bloc preparation look similar, it must be noted that the tissue oxygen environments in these preparations are significantly different (Okada et al., 1993; Funk and Gourine, 2018) (see below). Therefore, caution is necessary when evaluating the functional significance of the biphasic respiratory responses in these preparations. The time window of the calcium imaging in the present study corresponds to initial (∼4 min) excitatory responses to changes in external gas concentration.

It has been reported that ATP release from astrocytes in the ventral medulla is important in central chemosensory mechanisms (Gourine et al.,2005a,b, 2010; Marina et al., 2016; Rajani et al., 2017). This mechanism should work after the blockade of conventional neurotransmission by TTX or cocktail blockers and should enable glia-glia and glia-neuron interactions. This type of signaling could be present in the NTS. Therefore, it is possible that the response of cells in the present study may be secondly induced by TTX- or cocktail blockers-independent mechanisms. Future study using other blockers is required to clarify these suppositions.

Our study is the first report, to our knowledge, that the NTS includes cells that may be dually sensitive to hypercapnia and hypoxia. Such dually sensitive cells may be important in the detection of hypoventilation frequently seen in patients with hypoventilation syndrome (Chebbo et al., 2011), chronic obstructive pulmonary disease (Jacono, 2013) and sleep-disordered breathing (Sowho et al., 2014), who suffer from hypercapnic hypoxemia. This report could be the basis for a better understanding of the cardiorespiratory regulatory mechanisms of hypercapnia and hypoxia and could contribute to the elucidation of the pathophysiology of diseases with disturbed cardiorespiratory responses to hypercapnia and hypoxia.

### Technical Limitations

The calcium responses tended to gradually decrease with time mainly due to photobleaching by laser illumination. Thus, the response should be highest in the initial test. Although this tendency was observed in the effects of the first hypercapnic test on the following hypoxia test (Table 2), it was statistically not significant. We treated as responses those that indicated a rapid change (typically more than 5% within less than 5 s, see also Supplementary Table 1). However, it was also possible that a single short response could be induced by chance (but not by the stimulation).

In the standard solution and cocktail blockers solution, it was expected that an increase in fluorescence would be detected in neurons in association with the increased firing rate during CO_2_ exposure. However, this was not clear in the present study. We think that our sampling rate (3.3 Hz) might have been too low for the detection and/or a calcium transient associated with a single action potential in each neuron would be less than the detection level of our measurement system. However, this sampling rate was enough to detect astrocyte activity (Dallwig and Deitmer, 2002; Hartel et al., 2007; Okada et al., 2012; Stobart et al., 2018) as well as respiratory related neuronal burst activity in the ventral medulla (Ruangkittisakul et al., 2008; Onimaru et al., 2018). Considering recording time (5 min/test) and data size, we thought that 3.3 Hz was enough and appropriate to record astrocyte activity.

We used low K^+^ stimulation to identify astrocytes, although some astrocytes would not respond to this stimulation as discussed above. As another method of identification, sulforhodamine 101, which can be taken up specifically into astrocytes in the hippocampus and cortex (Schnell et al., 2012) might be applicable. However, this method has several drawbacks and could not be applicable to the identification of astrocytes in the medullary regions (Schnell et al., 2012; Hülsmann et al., 2017). For identification of cell types, it would be useful to compare soma size (Huxtable et al., 2010; Okada et al., 2012), although this remains for future study.

We used only newborn rats (P0–P4). As the number of astrocytes in the brain increases with development (Voigt, 1989; Botchkina and Morin, 1995), the functional importance would change with development. Thus, we should carefully compare the results in preparations from rats of different ages.

In the en bloc preparation, oxygen is supplied from the superfusate to the tissue via diffusion through the surface of the preparation, and there is a gradient in PO_2_ which is high on the surface and lower in the deeper region (Brockhaus et al., 1993; Okada et al., 1993; Funk and Greer, 2013). In the present study, we recorded cellular activities by calcium imaging using a confocal microscope. This method enabled us to visualize cells in the NTS region up to approximately 100 μm deep from the cut surface of the preparation (Onimaru et al., 2018). Indeed, the focal plane in our imaging was limited in this superficial region, where the tissue might be under hyperoxic condition (Brockhaus et al., 1993; Okada et al., 1993). Thus, NTS cells in the region analyzed can respond to a hypoxic stimulus, but the physiological significance of this sensitivity remains to be established.

## Conclusion

Calcium imaging in the NTS revealed that this region included cells that could respond to hypercapnic, hypoxic and both types of stimulation, and some of the cells were suggested to be astrocytes. These cells may possess a basic ability to act as a central gas sensor.

## Data Availability Statement

The raw data supporting the conclusions of this article will be made available by the authors, without undue reservation.

## Ethics Statement

The animal study was reviewed and approved by Ethics Committee for Animal Experiments of Murayama Medical Center.

## Author Contributions

HO and IY designed and performed the experiments, analyzed the data, and wrote the manuscript. IF and KT contributed to data acquisition and analysis. YO contributed to design of the experimentation and helped to draft the manuscript. All authors read and approved the final manuscript.

## Conflict of Interest

The authors declare that the research was conducted in the absence of any commercial or financial relationships that could be construed as a potential conflict of interest.

## Supplementary Material

Supplementary Video 1 Calcium imaging by Oregon Green of cell activity in the NTS in response to low K^+^ stimulation, corresponding to Figure 2.
