# Supplementary material for: Calcium Imaging Analysis of Cellular Responses to Hypercapnia and Hypoxia in the NTS of Newborn Rat Brainstem Preparation
Source: Front Physiol. 2021 Mar 25;12:645904. doi: 10.3389/fphys.2021.645904 (PMC8027497; doi:10.3389/fphys.2021.645904)
Supplement: Supplementary file 1 [file Data_Sheet_1.PDF]

SUPPL. TABLE 1. Characteristics of excitation in response to stimulations.

		N	Duration of excitation (s)				ΔF/F × 100			
**Standard**	Hypercapnia	49	31.3	±	33.7		12.1	±	7.9	
	Hypoxia	39	48.6	±	44.8		10.4	±	5.4	
	Low K^+	43	72.4	±	40.9	*** $	9.5	±	4.8	
**Cocktail**	Hypercapnia	38	28.7	±	31.9		10.5	±	5.2	
	Hypoxia	40	40.3	±	43.9		9.9	±	5.3	
	Low K^+	33	52.1	±	41.5	*	8.1	±	3.8	
**TTX**	Hypercapnia	34	22.9	±	24.4		8.7	±	5.1	
	Hypoxia	35	35.8	±	31.9		6.9	±	3.8	* $$
	Low K^+	30	69.8	±	53.7	*** $$	6.2	±	2.8	**
**FA**	Hypercapnia	24	33.2	±	38.0		7.5	±	3.5	*
	Hypoxia	18	42.2	±	44.4		7.3	±	3.4	
	Low K^+	12	45.4	±	41.2		6.1	±	3.3	*

Standard, in standard solution; Cocktail, in cocktail blockers solution; TTX, in 0.5 μM solution; FA, in 5 mM fluoroacetate + 0.5 μM TTX solution. Hypercapnia, cells that responded to hypercapnic stimulation (2% CO_2→8% CO_2); Hypoxia, cells that responded to hypoxic stimulation (95% O_2→0% O_2); Low K^+, cells that responded to low K^+ solution. Duration of excitation (s), duration that fluorescence intensity increased compared with the baseline. ΔF/F × 100, ΔF/F (percentage) of the peak value. Duration of peak (s) was analyzed in each group; **P* <0.05, ****P* < 0.001; compared with Hypercapnia and $*P* <0.05, $$*P* < 0.01; compared with Hypoxia (by one-way ANOVA followed by Tukey–Kramer multiple comparisons test). ΔF/F in each group did not differ significantly. Difference of ΔF/F in each stimulus condition was significant in some cases; **P* <0.05, ***P* < 0.01; compared with those in the standard solution; $$*P* < 0.01; compared with that in cocktail blockers solution (by one-way ANOVA followed by Tukey–Kramer multiple comparisons test).
