# Supplementary material for: Calcium Imaging Analysis of Cellular Responses to Hypercapnia and Hypoxia in the NTS of Newborn Rat Brainstem Preparation
Source: Front Physiol. 2021 Mar 25;12:645904. doi: 10.3389/fphys.2021.645904 (PMC8027497; doi:10.3389/fphys.2021.645904)
Supplement: Supplementary file 2 [file Data_Sheet_2.PDF]

Transient increase of C4 burst rate during hypoxic stimulation in the brainstem-spinal cord preparation

Methods

Experiments were performed with brainstem-spinal cord preparations from newborn (postnatal day 0-1) Wistar rats. The newborn rats were deeply anesthetized with isoflurane. The brainstem and spinal cord were isolated and superfused at a rate of 3.0 mL/min with the following artificial cerebrospinal fluid (in mM): 124 NaCl, 5.0 KCl, 1.2 KH_2PO_4, 2.4 $CaCl_2$, 1.3 $MgCl_2$, 26 $NaHCO_3$ and 30 glucose, equilibrated with 95% O_2 and 5% CO_2, pH 7.4, at 25-26°C. In most experiments, the preparations were cut transversely at a level just rostral to the anterior inferior cerebellar artery. For hypoxic stimulation (15 min), the superfusate was exchanged to 0% O_2 from 95% O_2 at 5% CO_2.

The initial data analyses were performed using the LabChart 7 Pro software program (ADInstruments, Castle Hill, Australia). The burst rate was calculated from 10 consecutive respiratory cycles. Data are presented as the mean ± SD. The significance of values was analyzed by a one-way ANOVA, followed by a Tukey-Kramer multiple comparisons test at a confidence level of $P < 0.05$ using the GraphPad InStat software program (GraphPad Software Inc., La Jolla, CA, USA).

Results

We examined C4 burst rate change in response to hypoxic stimulation in the brainstem-spinal cord preparation (n=7). The C4 burst rate was increased at 2-3 min after the start of hypoxic stimulation followed by a decrease: 3.7 ± 1.1/min in control, 8.4 ± 1.3/min at 2-3 min after hypoxic stimulation ($P < 0.001$, compared to control), and 4.2 ± 0.96/min after 15 min of the hypoxia. A typical example is shown in Suppl. Fig 1.

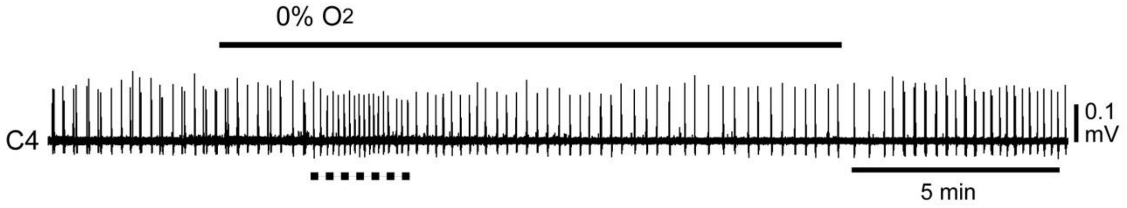

Suppl. Figure 1. Transient increase of C4 burst rate during hypoxic stimulation. C4 burst rate was increased at 2-3 min (dotted line) after the start of hypoxic stimulation (0% O_2) followed by a decrease.
